# Optical Coherence Tomography for Patients with Developmental Disabilities: A Preliminary Study

**DOI:** 10.3390/s21237940

**Published:** 2021-11-28

**Authors:** Kimberly Espinoza, Juri Hayashi, Yasushi Shimada, Junji Tagami, Alireza Sadr

**Affiliations:** 1 Dental Education in the Care of Persons with Disabilities (DECOD), Department of Oral Medicine, School of Dentistry, University of Washington, Seattle, WA 98195, USA; kmespino@uw.edu; 2 Biomimetics, Biomaterials, Biophotonics, Biomechanics & Technology (B4T) Laboratory, Department of Restorative Dentistry, University of Washington, Seattle, WA 98195, USA; jhayashi@uw.edu; 3 Cariology and Operative Dentistry Department, Graduate School of Medical and Dental Sciences, Tokyo Medical and Dental University, Tokyo 113-8549, Japan; shimada.ope@tmd.ac.jp (Y.S.); tagami.ope@tmd.ac.jp (J.T.)

**Keywords:** dental caries, developmental disability, radiology, optical coherence tomography, diagnosis

## Abstract

Dental radiographs are essential for diagnosis and treatment planning, but are sometimes difficult to acquire for patients with developmental disabilities (PDD). Optical Coherence Tomography (OCT) is a non-ionizing imaging modality that has the potential application as an alternative to dental radiographs for PDD. This study aimed to determine the feasibility of intraoral OCT imaging for PDD. Ten participants were recruited in the Dental Education in the Care of Persons with Disabilities (DECOD) Clinic to explore the utility of dental OCT. The prototype system (Yoshida Dental) creates in-depth and three-dimensional images of teeth. The participants indicated their degree of pain during imaging on the Wong-Baker FACES Pain Rating Scale, and the degree of discomfort after imaging on a visual analog scale. OCT can be used for patients with developmental disabilities with minimal levels of pain and discomfort, without ionizing radiation.

## 1. Introduction

Dental care is difficult for many, especially among people with developmental disabilities (PDD) [1,2,3,4,5,6]. This is a diverse population and includes individuals with cerebral palsy, Down syndrome, autism and intellectual disabilities, among other developmental conditions. The difficulty of tolerating dental care may stem from the impact of developmental conditions or co-occurring conditions that occur at a higher frequency in this population. For example, anxiety disorders are common among PDD and increase the risk of dental anxiety [7,8]. Specific challenges to dental care for PDD include dental anxiety and fears, movement disorders, swallowing disorders, hyperactive gag reflex, anatomical differences, communication disorders or differences, or difficulty following procedural instructions. Dental care difficulties are compounded by the discomfort often produced when taking dental radiographs, which are essential to the diagnosis and prevention of dental diseases. While exposure to dental X-rays does not cause pain, the pain and discomfort caused by dental radiography is typically attributed to imaging procedures. Pain and discomfort were reported for both traditional film and digital sensors, with and without the use of a holder [9,10]. A previous study on children reported that perceptions of pain from radiographic examinations were similar in intensity to dental injections [11]. It was even shown that the level of discomfort during radiographic imaging affects the accuracy of the diagnosis [12].

As many factors contribute to the difficulty to obtain high-quality radiographs for PDD, a variety of techniques are needed to support patients in obtaining the necessary diagnostic images. One technique is to obtain fewer images, such as a mini-series of one periapical image in each sextant instead of a full mouth series [13]. Another technique is to obtain different types of images, such as the use of a panoramic image or oblique lateral radiographs [14]. Other techniques include behavioral, pharmacological and physical facilitation techniques, each with potential benefits and limitations [15,16,17,18,19]. One such technique is to physically support the patient during radiograph exposure. Techniques involving “staying in the room” during X-ray exposure are not without controversy. There are legal and ethical responsibilities to both provide appropriate care to people with disabilities and keep the dental team safe. Health care providers, including dental professionals, are mandated under the Americans with Disabilities Act (ADA) to provide needed accommodations for patients with disabilities [20,21,22,23].

Keeping the dental team safe from ionizing radiation is essential. While the amount of ionizing radiation associated with the digital dental radiographs commonly used today is relatively low compared to the use of film radiographs, it is important to reduce the amount of radiation exposure as much as possible [24,25]. The standard for radiation safety is that the exposure to ionizing radiation should be “as low as reasonably achievable” (the ALARA principle) [26]. In keeping with the ALARA principle, if the exposure to ionizing radiation can be reduced, or in fact eliminated, using alternative imaging technologies, then these technologies should be explored [27]. An ideal imaging technology for PDD would not only have an excellent diagnostic power (i.e., sensitivity and specificity), but also be non-ionizing, in order to reduce exposure to both the patient and person assisting the patient during exposure. It would also produce real-time images with source–detector pairing to decrease the artifacts associated with movement. The real-time images produced would allow the provider to adjust the positioning of the device while obtaining the images to increase patient comfort.

In the past, light-based caries diagnostic systems were introduced [28]. Among them, optical coherence tomography (OCT) imaging technology has a great potential to meet the requirements mentioned above [29]. OCT is cutting-edge technology which can be used in a clinical dental setting without the risk of ionizing radiation to the patient or dental personnel. OCT works on a similar principle as ultrasonic imaging, but it has a higher resolution for hard tissue imaging. A light source is projected over the area of interest, and an in-depth image is reconstructed from the echo of the projected wave. In other words, OCT can construct images through the wave interference that occurs when backscattered light from a sample is coupled with a reference light. OCT visualizes differences in the tissue’s optical properties, which includes the effects of both optical absorption and scattering. In particular, “swept-source” OCT can construct images by the ultrahigh speed scanning of the time-encoded wavelength of a near-infrared laser. It was shown that this modality allowed for the non-invasive construction of tomographic images of biological substrates in a short period, almost in real-time.

OCT is an established medical imaging technique widely used to obtain high-resolution images of the anterior segment of the eye and the retina. It was shown that imaging teeth and non-metallic restorations using near infrared OCT may facilitate the clinical diagnosis of caries and evaluation of existing restorations [30,31,32]. The diagnostic power of OCT for caries was investigated in multiple studies [29,33,34,35]. OCT is a highly sensitive tool for monitoring early white spot lesions that was applied in clinical trials on remineralization [36,37,38]. It is suggested that diagnostic methods for caries should have at least a 0.75 sensitivity and over 0.85 specificity to be effective [39]. In one study on the clinical detection of proximal caries in unrestored teeth, OCT showed a sensitivity of 0.84 and specificity of 0.82, compared to 0.65 and 0.73 for bitewing radiographs, respectively [34]. When it comes to characterizing occlusal caries, OCT showed a significantly higher sensitivity at various clinical thresholds including enamel demineralization, cavitated enamel caries, and dentin caries compared to radiography, while specificity was not statistically different [33]. However, there are no commercially available dental OCT systems as of now and there are no studies reporting the use of OCT in the PDD population. This study aimed to determine the feasibility of OCT as an imaging technique for PDD.

## 2. Material and Methods

### 2.1. Participant Recruitment and Consent

Participants were recruited from the Dental Education in the Care of Persons with Disabilities (DECOD) Clinic, which serves adults with developmental and acquired disabilities. The inclusion criteria were adults with a documented developmental disability and ability to assent to study procedures as determined by a special care dentist. The exclusion criteria were patients who were likely to require immobilization to complete study procedures, and edentulous patients. Eligible patients and their care representatives received a recruitment flier in the clinic waiting area. Those interested, obtained information about the study from one of two study coordinators. The study coordinator obtained informed consent from the patients, or their legal guardians, if they elected to participate in the study. A pictorial consent form in addition to a traditional written consent form were used to aid in patient communication.

### 2.2. OCT Imaging Process

**Figure 1:**
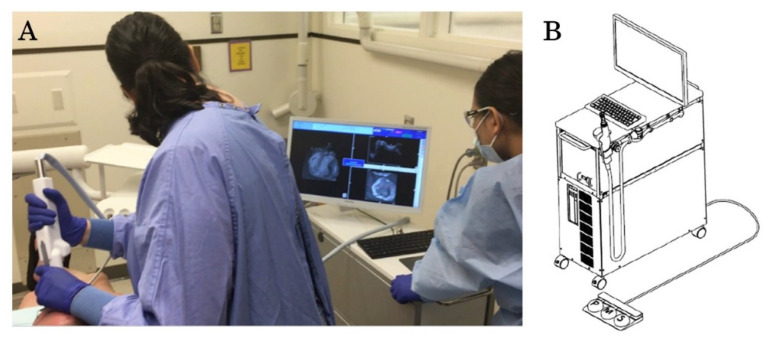
Clinical imaging using the prototype OCT system. The intraoral imaging probe is attached to the system. A preview image is available in real-time on the computer screen (**A**). A schematic overview of the OCT system. The recording and saving functions can be initiated using a foot pedal (**B**).

OCT images were obtained for each study participant using a prototype dental OCT system. This system uses a high-speed frequency swept laser light with a center wavelength of 1310 nm and a scan range of 140 nm at 50 KHz (Yoshida Dental Mfg., Tokyo, Japan). The *z*-axis resolution of the system is 11 µm in air. The OCT probe is similar in shape and size to a standard dental light curing unit or an intraoral digital scanner, with an anterior and posterior attachment. Anterior images were taken with the anterior probe attachment oriented toward the facial surfaces of the anterior teeth. Posterior images were taken with the posterior probe attachment oriented toward the occlusal surface of the posterior teeth. After wiping the tooth surface with a dental gauze, the provider (K.E.) used the OCT imaging probe to scan and record images. The provider was wearing standard personal protective equipment (PPE) without magnifying loupes and used dental overhead light during OCT imaging process. A dental assistant performed saliva suctioning and air blowing during imaging as needed. A rubber bite block was inserted to aid in patient comfort and mouth opening. As the handheld scanning probe was oriented over the area of interest, a preview was visible on the computer screen of a 10 × 10 mm region with an optical depth of 8 mm (x, y and z) (Figure 1). Once a satisfactory preview was observed, a 3D image was scanned with 400 × 400 × 2048 voxel resolution, which took 3.6 s per scan.

### 2.3. Data Collection and Analysis

All study participants completed brief pre and post discomfort and pain surveys. Two study coordinators administered these surveys and clarified study questions to patients when necessary. The surveys consisted of two questions each, one regarding discomfort and one regarding pain. The pre-survey asked participants about their pain and discomfort during their past dental X-rays. The post-survey asked participants about their pain and discomfort during the OCT imaging procedures. Each survey included a photo representation of a dental X-ray holding device (pre) or an OCT probe (post). For indicating pain, the pictorial Wong-Baker FACES Pain Rating Scale with a range of 0–10 was used. Participants pointed to the face symbol representing the level of pain they experienced and the corresponding number was recorded. For discomfort, a visual analogue scale (VAS) was used with a range of 0–100. Participants made a mark on the line of the VAS representing their amount of discomfort and this recording was measured.

This protocol was approved by the institutional review board for research on human subjects.

## 3. Results

A total of 10 participants completed the OCT imaging and pre and post surveys. The age range of participants was from 24 to 61 years old, and their dental X-rays within the past year were obtained. The participants had a diagnosed developmental disability, which included intellectual disabilities, cerebral palsy, Down syndrome or autism. One participant had a history of supported radiographs, which required a member of the dental team to stay in the room during X-ray exposure.

OCT images were obtained for all of the 10 participants. Eight of the participants were able to complete 100% of the planned images. Two of the participants were not able to complete OCT images of the second and third molars due to limited mouth opening. Subjectively, anterior images were easier for the study team to obtain than posterior images. This was due to easier access as well as the probe design. For posterior images, the inside of the probe and the mirror attachment frequently fogged, which inhibited obtaining the OCT image. Gently blowing air on the mirror and probe allowed the device to defog and the OCT image to be obtained. Asking participants to breathe through their nose helped to some extent, although nose breathing was difficult for many participants.

**Figure 2:**
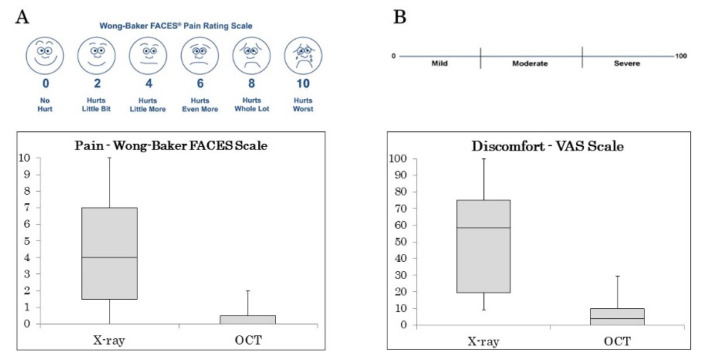
Statistical distributions of the Wong-Baker FACES scale pain scores (**A**) and VAS discomfort scale (**B**). Box plots are given for X-ray and OCT groups. The horizontal lines represent the medians and quartiles. The top and bottom of the vertical lines specify 1.5 times the interquartile range plus and minus the upper and lower quartiles, respectively.

Figure 2 presents the results of the Wong-Baker FACES Pain Rating Scale and discomfort VAS surveys. For X-rays, the pain ranged from 0 to 10 (median 4). For OCT images, the pain ranged from 0 to 2 (median 0). For X-rays, the VAS discomfort ranged from 9 to 100 (median 63.75). For OCT images, the VAS discomfort ranged from 0 to 29.5 (median 1.75).

**Figure 3:**
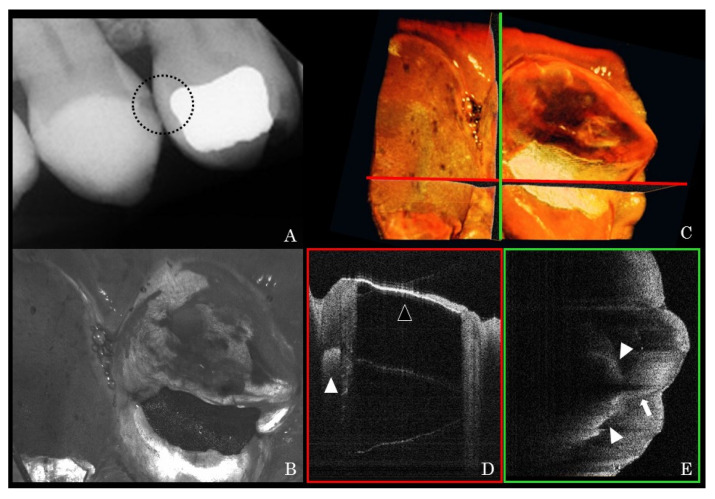
Representative bite-wing X-ray image of upper right premolars (**A**) and the corresponding OCT XY en-face image at the similar area (**B**). Mesial-distal and buccal-lingual cross-sectional images (**D**,**E**) at horizontal and vertical lines on the 3D image (**C**) were reconstructed from the OCT raw data. In the mesial-distal cross-section (**D**), the bright line at the occlusal surface (blank arrow head) indicates that OCT laser completely reflected at the amalgam restoration and could not obtain the inner image. The high signal intensity at the distal part of the first premolar indicates proximal caries (**D**,**E** bold arrow heads). In the buccal-lingual cross-section at the distal proximal part (**E**), the high signal line indicates vertical crack progress (bold arrow). Crack formation results in light scattering are observed as a high brightness boundary within seemingly sound dental tissue. These cracks are frequently observed at the marginal ridges of teeth with old occlusal amalgam restorations.

**Figure 4:**
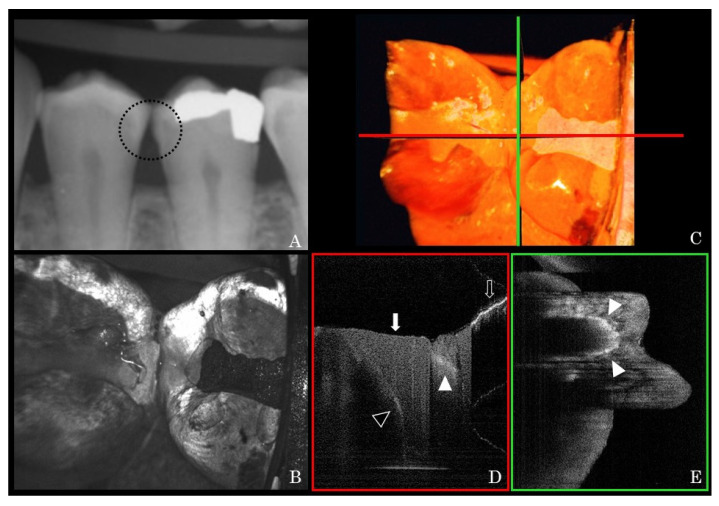
Representative bite-wing X-ray image of lower left premolars (**A**) and the corresponding OCT XY en-face image at the similar area (**B**). Mesial-distal and buccal-lingual cross-sectional images (**D**,**E**) at horizontal and vertical lines on the 3D image (**C**) were reconstructed from the OCT raw data. In the mesial-distal cross-section (**D**), in contrast to the bright line at the occlusal surface of amalgam restoration (blank arrow), OCT laser was able to penetrate into the resin composite restoration (bold arrow) and show the interfacial gap formation (blank arrow head). The high signal intensity at the mesial part of the second premolar, which indicates proximal caries, could be observed in the buccal-lingual cross-section at the distal proximal part (**D**,**E** bold arrow heads). This pattern of light scattering within proximal enamel suggests a cavitated proximal caries lesion, or a moderate caries lesion. The round pattern internal scattering resembles the boundaries of enamel surface breakdown.

**Figure 5:**
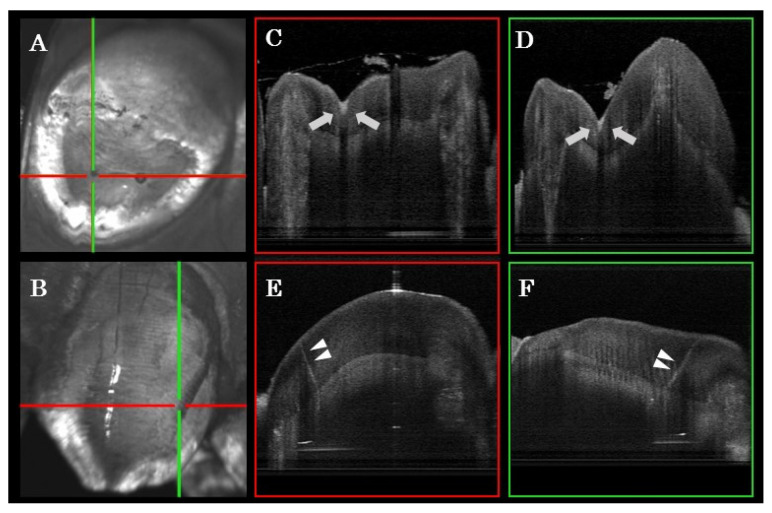
OCT XY en-face image of a lower premolar (**A**) and upper canine (**B**), with corresponding cross-sectional (XZ and YZ) images (**C**–**F**), indicated by horizontal (red) and vertical (green) lines on the en-face images. At cross point of the cross-sections of the lower premolar, a high signal intensity area at a pit and fissure part of the occlusal surface indicates early caries lesion (**C**,**D** arrows). This pattern of scattering at the base of occlusal fissures indicates initial enamel caries lesion without surface breakdown (no cavitation). At cross point of the cross-sections of the upper canine, a fine bright line indicates crack progress extending from the enamel surface into DEJ (**E**,**F** arrow heads).

Representative clinical OCT scans and counterpart dental X-ray radiographs are presented in Figure 3 and Figure 4. The high signal intensity at the interproximal area of posterior teeth indicated proximal caries (Figure 3D and Figure 4D). Unlike amalgam restorations that blocked OCT light (Figure 3D), the laser could penetrate into the resin composite restoration and indicate internal debonding (Figure 4D). Other noteworthy findings from the OCT images included early demineralization characterized at the base of occlusal fissures (Figure 5C,D) on a lower premolar. A high signal intensity area at a pit and fissure part of the occlusal surface indicates early caries activity. This pattern of scattering at the base of occlusal fissures indicates an initial enamel caries lesion without surface breakdown (no cavitation). Additionally, cracks penetrating the whole thickness of enamel on smooth surfaces were shown (Figure 5E,F) on an upper canine tooth. A fine bright line indicated crack progress extending from the enamel surface into DEJ. These hairline cracks progress across the whole thickness of enamel and may not pose a risk to caries if not in the plaque stagnation area.

## 4. Discussion

The results of current study indicated that OCT is a feasible imaging technique causing minimal pain and discomfort to the participants. OCT images produced no pain or mild pain for all participants, with the majority reporting no pain.

To the best of our knowledge, this is the first report on the use of OCT for clinical dental imaging in any population with special health care needs, including PDD. Currently, there is no dental OCT system commercially available for clinical use, and a prototype dental OCT system was used in this study. The OCT intraoral probe is operated by the provider and does not require the patient to keep still, hold something in their mouth or bite on a device for a long period of time. Compared to prior studies that used 20 KHz sources, the current 50 KHz OCT could produce 3D volumes in a shorter time (3.6 s) and provide a real-time 3D preview. Imaging time is critical for many PDD patients, especially those with movement disorders or other difficulties with staying still during imaging procedures. Motion artifacts increase with slower imaging systems; however, the high-speed feature of this system was particularly valuable. The current OCT system obtains the 3D volume as 400 independent 2D cross-sections (B-scans) across the area of scan; therefore, PDD movements during the 3.6 s generally do not affect the quality of 2D cross-sections, even though the 3D volume may appear distorted.

A recent report showed that the dynamic slicing of 3D OCT images enabled the chairside evaluation of spatial features, such as the cavitation dimensions and caries spread at DEJ within a clinically realistic time [33]. This 3D strategy also demonstrated an excellent reliability among multiple examiners. Similar to all medical imaging systems, the visual interpretation of OCT images requires appropriate training and calibration [40]. OCT is capable of picking up microscopic changes, microcracks and microporosities in tissues to a a level comparable to a destructive high-resolution microscopic examination [41].

On the other hand, OCT imaging has considerable limitations that should not be overlooked. Imaging depth is limited by the optical properties of an object [42]. The near infrared light cannot penetrate metallic restorations, and is restricted from reaching subgingival interproximal areas for primary or secondary caries imaging. In addition, maneuvering the current prototype wand in patients with limited mouth opening or in tight spaces, such as the buccal surface of the last molar, can be difficult. The imaging size of 10 mm in x and y dimensions on a surface would also mean that several shots would be required to cover various surfaces or multiple teeth with the current technology.

There were also several challenges in designing a study to obtain perceptions of pain and discomfort from PDD. The first challenge was in obtaining informed consent for the study. Some PDD patients do not have legal competency to provide informed consent. In these cases, a legal guardian provided the informed consent. To avoid the ethical implications of performing research procedures on PDD without the legal competency to consent, the inclusion criteria specified that participants must be able to assent to care. In other words, they must understand and agree to the study procedures. If any participants were to have withdrawn their assent, the study procedures would have been discontinued. It is possible that the consideration of assent concerns during the study would lead the provider taking the OCT images to be more cautious with assuring patient comfort. This has the potential to bias study results. It was also anticipated that some PDD would have difficulty understanding a traditional written informed consent form. For this reason, a pictorial consent form was developed to help participants understand study procedures. Most participants preferred the pictorial consent form over the written consent form. The requirement for patient assent and the use of a pictorial consent were strengths of this study.

The second challenge was in recording participants’ perceptions of pain and discomfort. It was anticipated that some PDD would have physical factors, cognitive factors, or communication disorders or differences that would make it difficult to fill out a written survey. The Wong-Baker FACES Pain Rating Scale and VAS were commonly used as simple and validated scales for measuring pain and discomfort [43,44]. Using these tools allowed study participants to effectively answer study questions. In some cases, participants asked for clarification about study questions. The study coordinator clarified participant questions. Some study participants preferred to have a caregiver or guardian in the room during study procedures. In some cases, the caregiver or guardian also helped with communication. While it is possible that the clarifications from the study coordinator and communication supports from caregivers or guardians introduced bias into survey results, this effect is likely to be minimal and similar for questions related to X-rays and OCT. It was hypothesized that some PDD patients were more likely to give positive responses to questions as a learned behavior, a phenomenon known as acquiescence [45]. It is possible that the PDD patients in this study would be biased toward giving responses they felt would be positive for the study team. Having a study coordinator who was not part of the clinic staff helped mitigate this concern. Additionally, the experience and reporting of pain may be affected by many factors, including physical, psychological and cognitive factors as well as communication factors, prior pain experiences, fear and anxiety [46].

A third challenge was the potential for recall bias related to the survey questions on X-ray radiography. The design of this preliminary study with a limited sample size was not a randomized controlled trial, and the claim of a cause-and-effect relationship would be difficult to prove. In addition, radiographs were not taken as part of the study in order to avoid unnecessary exposure to ionizing radiation. Instead, participants were asked about their past X-ray experiences. Many factors can affect the recall of acute painful experiences [47,48]. It is possible that PDD patients remembered more vividly their most negative radiography experience, and it is possible that the patients gave responses that were favorable to the study team. The problem of recall bias could be avoided if the pre-survey was administered immediately after radiographs were taken; however, this was not carried out due to the complexity of coordinating study procedures with patient care procedures. The memory of a painful procedure can be different from experiencing it; therefore, the interval between the X-ray radiography and OCT, which was up to a year in this study, might be the cause of bias [49]. This is indeed a limitation of this study. In addition, because of the retrospective nature of the X-rays, it was not possible to ensure that the OCT images were taken from the exact same teeth and under the exact same conditions.

Obtaining high-quality radiographic images for PDD is often difficult. The reasons for this are multifactorial, and include pain and discomfort and movement artifacts. The consequences of not obtaining diagnostic dental images for PDD is significant, especially among PDD patients who have difficulty communicating their symptoms. The current limited clinical study with a small number of participants, suggested that OCT imaging technology has a feasibility to meet the requirements to become a diagnostic imaging tool without ionizing radiation for PDD patients, and with minimum discomfort and pain.
